# Serum Antibodies against CD28– A New Potential Marker of Dismal Prognosis in Melanoma Patients

**DOI:** 10.1371/journal.pone.0058087

**Published:** 2013-03-06

**Authors:** Rebecca Körner, Klaus-Dieter Preuss, Natalie Fadle, Darius Madjidi, Frank Neumann, Lennart Bergeler, Stefan Gräber, Cornelia S. L. Müller, Frank Grünhage, Michael Pfreundschuh, Frank Lammert, Thomas Vogt, Claudia Pföhler

**Affiliations:** 1 Department of Dermatology, Saarland University Hospital, Homburg/Saar, Germany; 2 Department of Internal Medicine I and José Carreras Centre for Immuno and Gene Therapy, Saarland University Hospital, Homburg/Saar, Germany; 3 Institute for Medical Biometry, Epidemiology and Medical Informatics, Saarland University Hospital, Homburg/Saar, Germany; 4 Department of Internal Medicine II, Saarland University Hospital, Homburg/Saar, Germany; Carl-Gustav Carus Technical University-Dresden, Germany

## Abstract

**Background:**

Autoantibodies against CD28 have been found in patients with autoimmune and atopic diseases. These antibodies may act as superagonists and activate T cells but may also be antagonistic or induce immunosuppressive effects by activating regulatory T cells. Autoimmunity in melanoma patients has been discussed controversially.

**Objective:**

We investigated 230 melanoma patients for the occurrence of CD28 antibodies and the effect of the latter on overall and progress-free survival.

**Methods:**

We constructed an ELISA assay to measure CD28 serum antibodies. 230 patients with melanoma and a control-group of 625 patients consistent of 212 patients with virus hepatitis b or c, 149 patients with allergies, 78 patients with psoriasis, 46 patients with plasmocytoma and 140 healthy blood donors were investigated for the occurrence of CD28 antibodies.

**Results:**

CD28 abs occur at a higher percentage in patients with melanoma and in patients with viral hepatitis than in other groups investigated (p<0.001). Occurrence of CD28 abs is significantly higher in patients receiving interferons independent from the underlying disease (p<0.001). *In vitro* CD28 serum antibodies have an inhibitory effect on the CD28 receptor as they lead to reduced stimulation of Jurkat cells. Presence of CD28 was correlated with a higher risk of dying from melanoma (p = 0.043), but not with a significantly shortened overall survival or progression-free survival.

**Conclusion:**

Interferon therapy appears to induce the production of CD28 abs. In light of reports that these CD28 abs induce immunosuppressive Tregs and – as our data show – that they are inhibitors of CD28 receptor mediated stimulation, the continuation of therapies with interferons in melanoma patients developing CD28 antibodies should be critically reconsidered, since our data indicate a worse outcome of patients with CD28 abs.

## Introduction

The efficient activation of naive T cells by antigen-presenting cells (APC) requires the engagement of both the T cell receptor (TCR) and the costimulatory molecule CD28 [1]. On the surface of T cells, CD28 and cytotoxic T lymphocyte antigen 4 (CTLA-4) maintain a balance between immune activation and tolerance [2]. Blocking of CTLA-4 by targeted drugs such as ipilimumab results in an unopposed activation of CD28 resulting in immunostimulation and a breakdown of tolerance [3].

CD28 superagonistic antibodies are able to activate T cells without the need of further signals. As a matter of principle, these superagonists may activate effector T cells, but they seem to induce mainly immunosuppressive effects by activating *bona fide* CD4+CD25+ Treg cells or may be inhibitors, depending on the kind of antibodies.

Autoantibodies against CD28 have been found in patients with atopic diseases, e.g. allergic rhinitis and asthma [4]. It was assumed that these antibodies stimulate T cells and may play an important role in chronic allergic inflammation, as sera from patients with atopic dermatitis containing CD28 abs were able to stimulate T cell proliferation *in vitro* [4]. Lühder *et al.* demonstrated two groups of monoclonal CD28 abs: those abs that provide the costimulation to T cells concomitantly exposed to a TCR-mediated signal (“conventional” mAb), and those (“superagonistic”) mAbs that fully activate primary resting T cells both *in vivo* and *in vitro* in the absence of signal 1 [5].

Immune dysfunction is an early event in cancer development and expands with progression to metastatic disease [6]. Critchley-Thorne *et al.* investigated interferon (IFN) signalling in patients with breast cancer, melanoma and gastrointestinal cancer [7]. The authors showed that IFN-α-induced signalling was reduced in T and B cells from all three cancer patient groups [7]. The same working group investigated signalling pathways in T lymphocytes from patients with metastatic melanoma [8]. They showed by using peripheral blood lymphocytes from melanoma patients that one third of the patients was IFN-responsive, whereas the remaining two-thirds were only low-responsive [9]. Furthermore, T cells from low-IFN-responsive melanoma patients exhibited a decreased expression of activation markers [9]. Stimulation of these T cells with anti-CD3/CD28 antibodies lead to reduced survival of the cells, demonstrating that an impaired T-cell-function in combination with defects in IFN-signalling represent important mechanisms of immune dysfunction in cancer [6], [9].

The occurrence of CD28 abs in melanoma patients has not been investigated so far, but it is likely that CD28 abs play an important role in the complex scenario of immune activation and tolerance in melanoma similar to differential expression of CD28 itself on T-lymphocytes during immunomodulating therapy [10]. We therefore conducted this retrospective study in which we investigated the prevalence of CD28 serum abs in melanoma patients in comparison to several control groups.

## Materials and Methods

### 1. Study Participants

Serum samples from 230 patients with malignant melanoma, 212 patients with viral hepatitis B or C, 149 patients with hayfever/allergic asthma or insect venom allergy, 78 patients with psoriasis vulgaris, 46 patients with multiple myeloma and 140 healthy blood donors were investigated for the presence of CD28 abs. The study was approved by the local ethics committee and carried out in compliance with the Helsinki declaration. All patients and controls gave written informed consent.

### 2. Melanoma Patients

Two-hundred and thirty patients with melanoma (age range 22–88 years, mean ± SD 59.65±15) were enrolled in the study (male, n = 123; female, n = 107). According to the 2009 American Joint Committee on Cancer tumour classification (AJCC) 62 melanoma patients were in stage I, 59 in stage II, 79 in stage III and 28 in stage IV at the time of blood sampling. The stage of 2 patients could not be determined because of unknown thickness of primary melanoma.

Tumour thickness of primary melanoma ranged from 0.4 to 18 mm (mean 2.5 mm).

Further patient and tumour characteristics are shown in table 1. Observation time varied from 0 to 4108 days (median 602 days). One-hundred and twenty of 230 melanoma patients (53%) received low- or high-dose-interferon therapy.

**Table 1:** Detailed data of melanoma patients.

		Patients 230 (100%)
**Gender**	male	123 (53.5%)
	female	107 (46.5%)
**Median age/years (range)**		60 (22–88)
**Stage of disease (AJCC 2009)**	IA	4 (1.7%)
	IB	58 (25.2%)
	IIA	29 (12.6%)
	IIB	20 (8.7%)
	IIC	10 (4.3%)
	IIIA	18 (7.8%)
	IIIB	34 (14.8%)
	IIIC	27 (11.7%)
	IV	28 (12.2%)
	Unknown	2 (0.9%)
**Tumor type**	SSM	71 (30.9%)
	NMM	94 (40.9%)
	LMM	5 (2.2%)
	ALM	13 (5.7%)
	AMM	28 (12.2%)
	UCM	8 (3.5%)
	Others	11 (4.8%)
**Median tumor thickness/mm (range)**		2,5 (0–18)
**Clark Level**	I	1 (0.4%)
	II	12 (5.2%)
	III	43 (18.7%)
	IV	130 (56.5%)
	V	14 (6.1%)
	Unknown	30 (13%)
**CD28 abs**		42 (18.3%)

SSM = superficial spreading melanoma, NMM = nodular malignant melanoma, LMM = Lentigo maligna melanoma, ALM = acrolentiginous melanoma, AMM = amelanotic melanoma, UCM = unclassified melanoma, others = melanoma of the mucosa or uvea.

### 3. Hepatitis Patients

In total, 212 patients with persistent or resolved chronic viral hepatitis B or C were investigated. Serum sampling was performed under therapy with IFN-α or shortly thereafter. One hundred thirty-nine (65.6%) patients were male, 73 (34.4%) were female (age range 22–79 years, mean 49.2±11.6 years).

### 4. Other Control Groups

The psoriasis group included 56 male and 22 female patients. Thirty-five (44.9%) of these patients suffered also from psoriasis arthritis. Furthermore, 72 male and 77 female patients with IgE-mediated allergies such as hayfever/allergic asthma bronchiale or insect venom allergy were investigated as well as 46 patients with plasmocytoma and 140 healthy blood donors.

### 5. Recombinant Expression of Human CD28 and Establishing of an ELISA for the Detection of Circulating Human CD28 Antibodies

**Figure 1:**
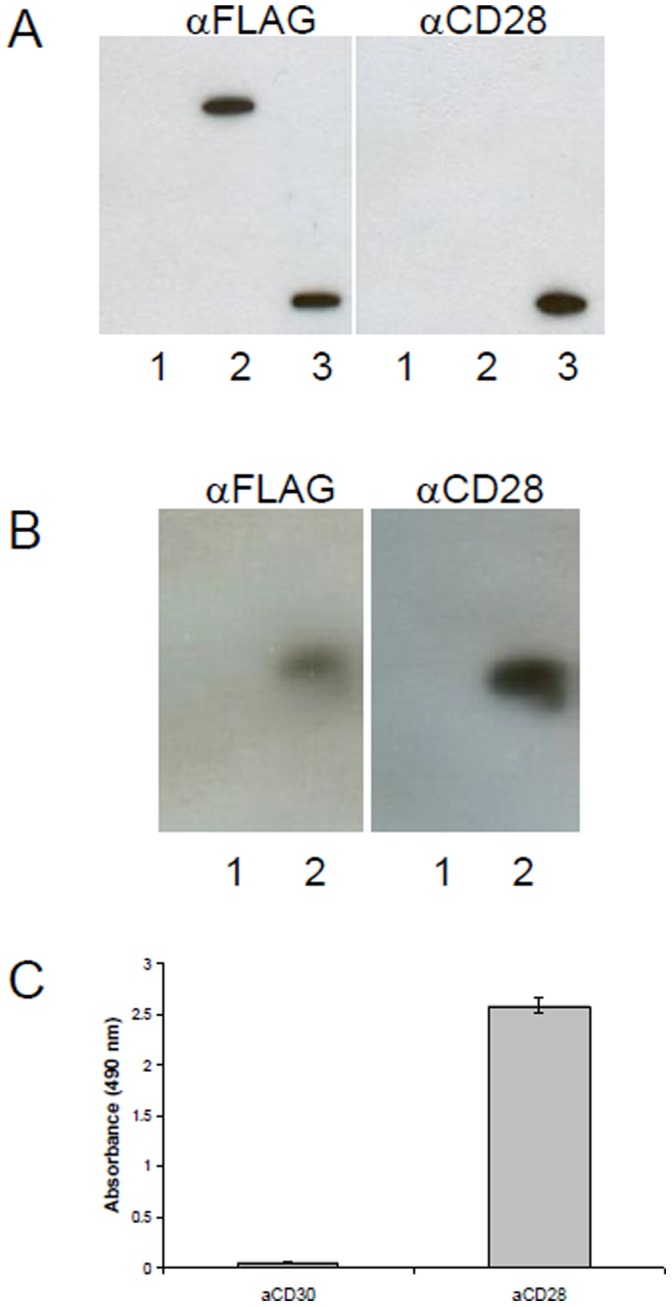
Analysis of recombinant CD28 expression in HEK cells. (A) Coimmunoprecipitation. Recombinant HEK cells were lysed with lysis buffer, and 200–500 µl of cell lysate was incubated with rabbit αFLAG antibody at 4°C for 2 hours, then 20 µl of protein A agarose slurry (GE Healthcare) was added for another 2 hours. The beads were washed three times with at least 10 volumes of lysis buffer before resolving by SDS-PAGE. Detection was done either with mouse αFLAG or mouse αCD28. As control HEK293-SLP2-FLAG was used. 1: HEK293 lysate, 2: HEK293-CD28-FLAG lysate, HEK293-SLP2-FLAG lysate. (B) Westernblot. Cells were lysed and analysed by immunoblot using αFLAG or αCD28 antibodies. 1: HEK293 lysate, 2: HEK293-CD28-FLAG lysate (C) Elisa. Recombinant CD28 is recognized by a commercial αCD28 mAb. HEK293-CD28-FLAG lysate is coated on NUNC maxisorp via FLAG-tag. Detection was done with 1: αCD30 or 2: αCD28.

**Figure 2:**
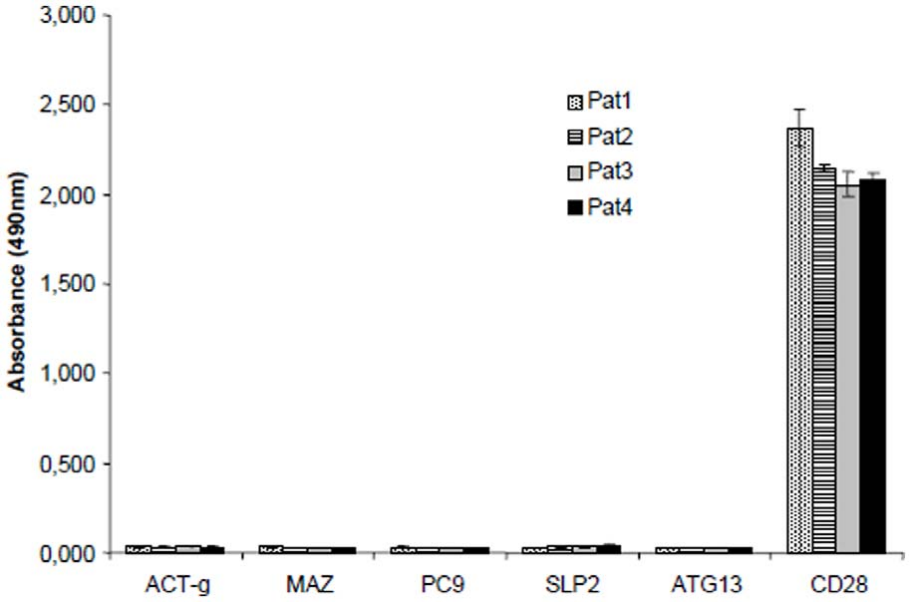
Specificity of the CD28 autoantibody Elisa. Human recombinant proteins were immobilized and measured by Elisa as described in the methods part. Sera from 2 patients were used. No unspecific signal was detected. ACT-g: actin-γ, MAZ: myc-ass. zinc finger protein, PC9: pyruvate-carboxylase 9, SLP2: stomatin-like protein 2, ATG13: autophagy related 13 homolog.

**Figure 3:**
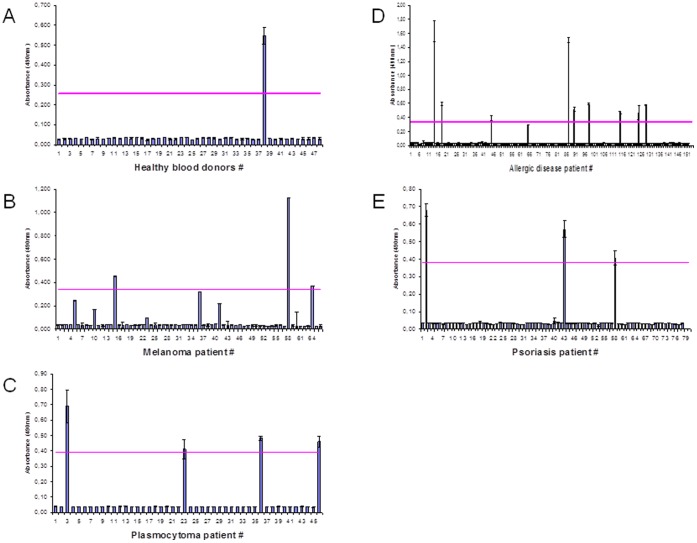
Occurrence of CD28 abs in different diseases. CD28-antibodies in the serum of healthy blood donors (A), patients with plasmocytoma (B), melanoma (C), psoriasis vulgaris (D) and allergies (E). Line: mean value (MV), dashed line: MV ±3 SD.

The CD28-antibody ELISA was established using recombinant full-length human FLAG tagged CD28. Full-length CD28 was amplified from activated Jurkat cell line DNA with primers introducing EcoRV restriction sites at both ends of the fragment and removing the stop codon (sense: GATATC ATG CTC AGG CTG CTC TTG GC; antisense: GATATC GGA GCG ATA GGC TGC GAA G). This fragment was cloned in pSfi-FLAG receiving an expression clone producing human CD28 with a FLAG-tag at the C-terminal end [11]. This clone was used to transfect HEK293 cells which are CD28 negative. After 3 days cells were lysed and checked for the expression of recombinant human CD28-FLAG. A NUNC maxisorb plate was coated with mouse anti-FLAG antibodies (Sigma, Munich, Germany) which were used for immobilizing the recombinant CD28-FLAG present in 10 µg of total cell extract. In detail, coating was done with anti-FLAG (1∶2500, 4°C, 16 h, Sigma, Munich, Germany) followed by blocking with 1.5% gelatine in washing buffer (1 h, RT) and incubation with the amount of recombinant CD28-FLAG protein which is present in 10 µl of total cell extract (1 h RT). Between each step intensive washing with TBS/0.1% Tx100 was done. Human serum was added at 1∶100 for 1 h at RT, followed by anti-human-IgG-Biotin (1∶2500, 1 h, RT) and Strep-POX (1∶50000, 1 h, RT). Development with OPD was done for 10 min at RT, stopped with HCl and measured at 490 nm. The specificity of the ELISA was checked using CD28 and other human proteins which were available in our lab and which were produced by the same procedure. No cross reaction was observed as shown in Figure 1 and 2. Sera from healthy blood donors were used and screened for the presence of CD28 abs (Fig. 3A). From these data cut-off was defined as mean value +3 standard deviation (MV +3SD). Samples with an absorbance lower than MV +10 SD were defined as negative; samples with an absorbance higher than MV +10 SD were defined as positive (Fig. 3).

### 6. Jurkat Experiments

Jurkat T cells (DSMZ number ACC282) were cultured in RPMI 1640 and tested for CD28 antigen expression using CD28 mAb (clone 15E8 IgG2b).

Human CD28 autoantibodies were purified from patients’ serum by affinity column chromatography. Recombinant expressed human Flag-tagged CD28 antigen in pSfi vector was purified from HEK293 cells, immobilized on sepharose beads and used for purification of the autoantibody. Elution was done with PBS pH 3.0 followed by neutralisation and dialysis. Purified autoantibody was checked for integrity by gel electrophoresis.

Jurkat cells (4×10^4^ cells/ml, 200 µl/well) were co-incubated in flat-bottom 96-well plates (Nunc) with purified human CD28 autoantibody (1 µg/ml) or with diluted patients serum (1∶10 in PBS) for 3 days at 37°C/5% CO_2_ followed by EZ4U cytotoxicity assay (Biomedicagroup, Vienna, Austria) according to the manual. Measuring was done on a micro-plate reader (Wallac Victor2, PerkinElmer, Rodgau, Germany) at 450 nm.

For a competition assay Jurkat cells were seeded (4×10^4^ cells/ml, 200 µl/well) in an uncoated flat-bottom 96-well plate. Arranged mixtures of mouse CD28-mAb and human CD28 autoantibody (0.5 µg mouse CD28-mAb mixed with increasing amounts (0.5 to 20 µg) of human CD28 autoantibody purified from patient serum) were added and incubated for 2 days at 37°C/5% CO_2_ followed by EZ4U cytotoxicity assay.

### 7. Thyreoglobulin (TG) Antibodies

TG antibodies were analysed using a commercial test (Varelisa TG antibodies, Phadia, Freiburg, Germany). The assay was performed as described in the manual. Each serum was analysed in duplicate.

### 8. ANA Test

Antibody assays were performed on sera collected from patients early in their presentation and stored at –20°C. The presence of ANA for all sera was confirmed concurrently in our laboratory by indirect immunofluorescence on monolayers of human larynx epidermoid carcinoma cells (HEp-2) (BioRad, Muenchen, Germany) at a screening dilution of 1∶40. A titre ≥40 was considered as positive. Each serum was analysed in duplicate.

### 9. Statistical Analysis

Dependencies in cross-classified tables were tested with Chi-square-test.

Progression-free survival was calculated as the time from the date of serum sampling until progression, the first relapse after obtaining a remission or death without relapse. Patients who did not relapse were censored at their last follow-up visit.

Overall survival calculated as the time from the date of serum sampling until death. Patients not dying were censored at their last follow-up. The Kaplan-Meier method was used to estimate the survivor function distributions, and the log-rank test was used to test for differences between survival curves. p<0.05 was considered to be significant. Cox regression models were used to investigate the association of the presence of auto-antibodies with overall or progress-free survival, whereas CD28 abs were considered to be a time dependent factor.

Statistical analysis was performed with SPSS 17 for Windows (SPSS GmbH, Munich, Germany).

## Results

### 1. Elisa for CD28 Autoantibodies

For the detection of CD28 autoantibodies in human serum an Elisa was constructed. Full-length human CD28 cDNA was amplified from Jurkat T cells and used for recombinant expression expression of human CD28 protein in HEK293 cells (which were negative for CD28). Recombinant CD28 protein was checked for integrity (Figure 1) and total cell extract was used as a coat in the CD28 Elisa. Specificity of the Elisa was controlled using sera from melanoma patients and other recombinant human proteins produced by the same procedure and available in our lab (Figure 2).

### 2. CD28 Autoantibodies

Presence of serum CD28 abs correlated significantly with the type of disease and was higher in patients with melanoma (42/230 = 18.3%) and patients with viral hepatitis (45/212 = 21.2%) (p<0.001, Chi-square-test) compared to patients with allergies (11/149 = 7.4%), multiple myeloma (4/46 = 8.7%), psoriasis (3/78 = 3.85%) or healthy blood donors (2/140 = 1.4%) (Figure 3).

### 3. Melanoma Patients

There was no correlation between the prevalence of CD28 abs and the stage of the disease (p = 0.588).

Fourteen (33.3%) of the 42 CD28 abs-positive patients were in stage III. However, no correlation was found between IFN therapy and the occurrence of CD28 abs in melanoma patients alone (p = 0.755). Twenty-one out of 42 patients with CD28 abs were treated with IFN, whereas 21 were not. There was also no correlation between the occurrences of CD28 abs in patients who received high- or low-dose-IFN-therapy. However, 13 of 21 patients (61.0%) under IFN treatment with CD28 abs suffered from progressive disease shortly after or during IFN therapy, while only 40 of 99 (40.0%) of the patients under IFN treatment without CD28 abs experienced progression. There was also no difference in CD28 antibody prevalence between male and female patients: 23/42 (54.8%) patients with CD28 abs were male and 19 (45.2%) were female (p = 0.5).

Furthermore, neither the type of primary melanoma (p = 0.211) nor its Clark Level (p = 0.240) correlated with the presence of CD28 abs, while 52% of the patients with CD28 abs had a primary melanoma with a depth of penetration according to Clark Level IV or V.

### 4. Overall Survival (OS) in Melanoma Patients

**Figure 4:**
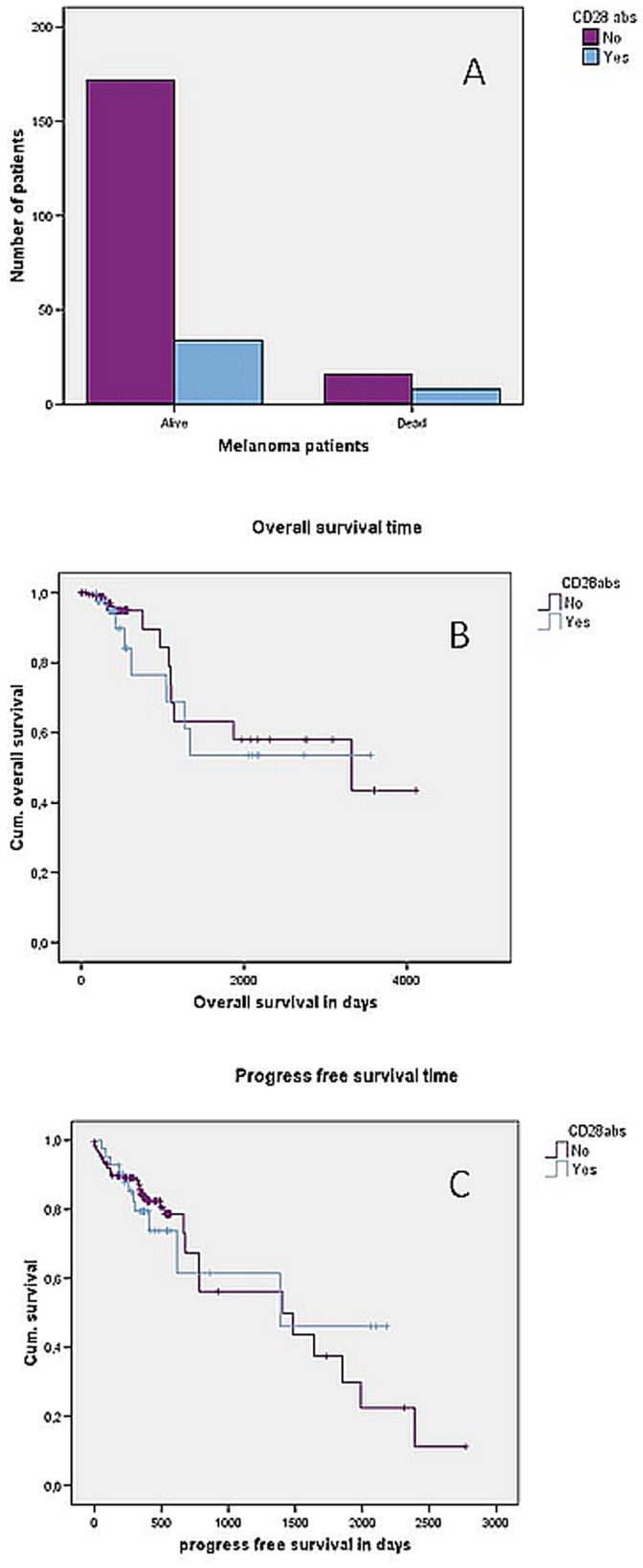
CD28 abs and death risk, overall survival and progress-free survival. CD28 abs and death risk (**A**). Distribution of patients with or without CD28 abs according to whether patients died from melanoma or not (p = 0.043, chi-square test), n = 230; **CD28 abs and overall survival** (**B**). Kaplan-Meier curve showing correlation between overall survival and occurrence of CD28 abs in melanoma patients (p = 0.559, Log-rank test), n = 230; **CD28 abs and progress-free survival** (**C**). Kaplan-Meier curve showing correlation between progress-free survival and occurrence of CD28 abs in melanoma patients (p = 0.952, Log-rank test), n = 230.

We found a significant correlation between the occurrence of CD28 abs and death of melanoma patients during the observation period. Twenty-four of 230 patients (10.4%) died of melanoma during the study period, and eight of them (33.3%) had CD28 serum abs (p = 0.043; Chi-square test, Figure 4A). However, we found no correlation between OS and CD28 abs (p = 0.559, Log-Rank test), but a tendency for a longer OS in patients without CD28 abs (Figure 4B). Mean OS for patients with CD28 abs was 2294 days in comparison to 2705 days for patients without antibodies. Median OS for patients without antibodies was 3322 days and was not reached for patients with CD28 antibodies because at the end of the observation period there were still more than 50% of these patients alive.

Cox regression model showed no differences in results regardless of whether the occurrence of CD28 was considered to be a time-dependent factor or not.

### 5. Progression-free Survival (PFS) in Melanoma Patients

There was no significant correlation between PFS and CD28 abs (p = 0.952, Log-Rank test). Mean progression-free survival was equal in both groups with 1364 days for patients without antibodies and 1359 days for patients with CD28 abs. We found no difference in median PFS. Median PFS was 1406 days for CD28 abs-negative patients and 1387 days for CD28 abs-positive patients (Figure 4C).

There were no differences in results regardless of whether CD28 was considered to be time-dependent or not.

### 6. Seroconversion in Melanoma Patients

We were able to measure the emergence of antibody titres in 6 patients over a period of several years. All patients were CD28 antibody negative at baseline. Four of these 6 patients showed a seroconversion during the observation period. Seroconversion was associated with a progression of the disease in all cases and all 4 patients died of melanoma.

**Figure 5:**
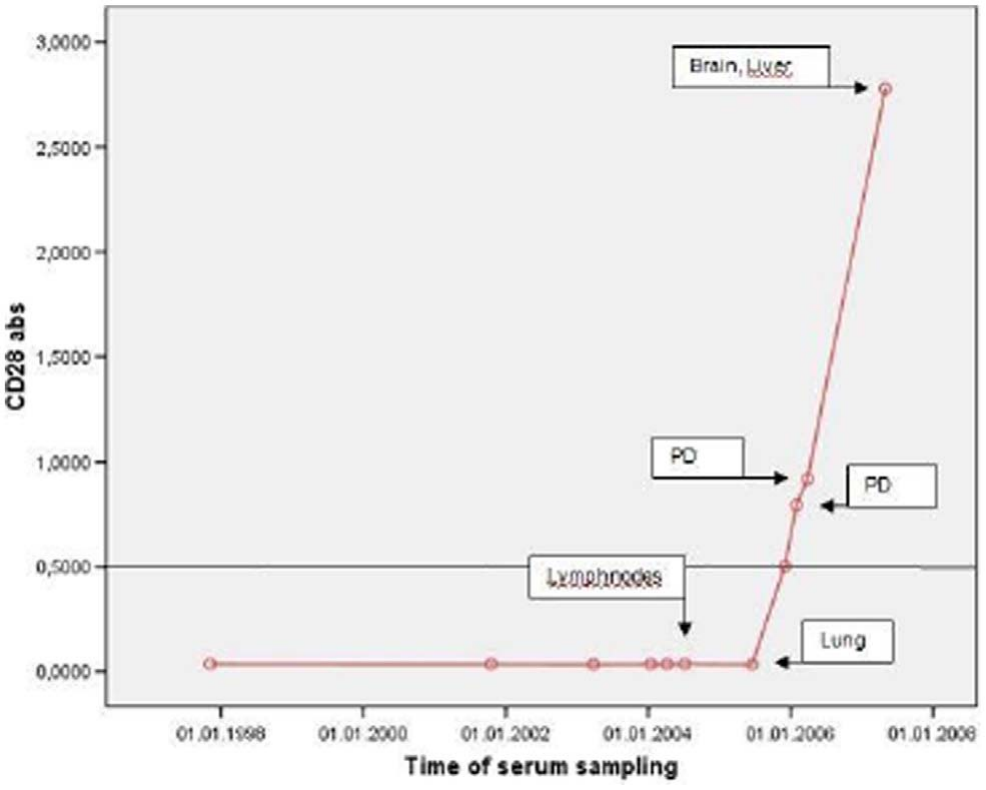
Seroconversion with progress of disease. CD28 abs measured in one female patient during a period of more than 10 years in association with the course of the disease.

In one female patient the course of CD28 antibody could be followed over for more than 10 years. In this patient a facial melanoma was diagnosed in 1996. At that time she did not have CD28 serum abs. Lung metastases were diagnosed June 2005, when CD28 abs were still not detectable. However, in December 2005 with progression lung metastases in January 2006 and March 2006 a continuous rise of CD28 abs titer with a maximum at diagnosis of brain and liver metastases in May 2007 was observed. The patient died shortly after from metastatic disease in May 2007 (Figure 5).

### 7. Thyreoglobulin (TG) Antibodies and Anti-nuclear Antibodies (ANA) in Melanoma Patients

Thirty of 230 (13.4%) melanoma patients had TG abs and 10 of 230 (4.34%) ANA. Both did not correlate with the occurrence of CD28 abs (p = 0.438 and p = 0.356, Chi-square-test). There was no significant correlation between PFS and the occurrence of TG abs or ANA (p = 0.333 and p = 0.946, Log-rank test), nor was there a significant correlation between OS and the occurrence of TG abs or ANA (p = 0.556 and p = 0.979, Log-rank test).

### 8. CD28 Antibody Titres

**Figure 6:**
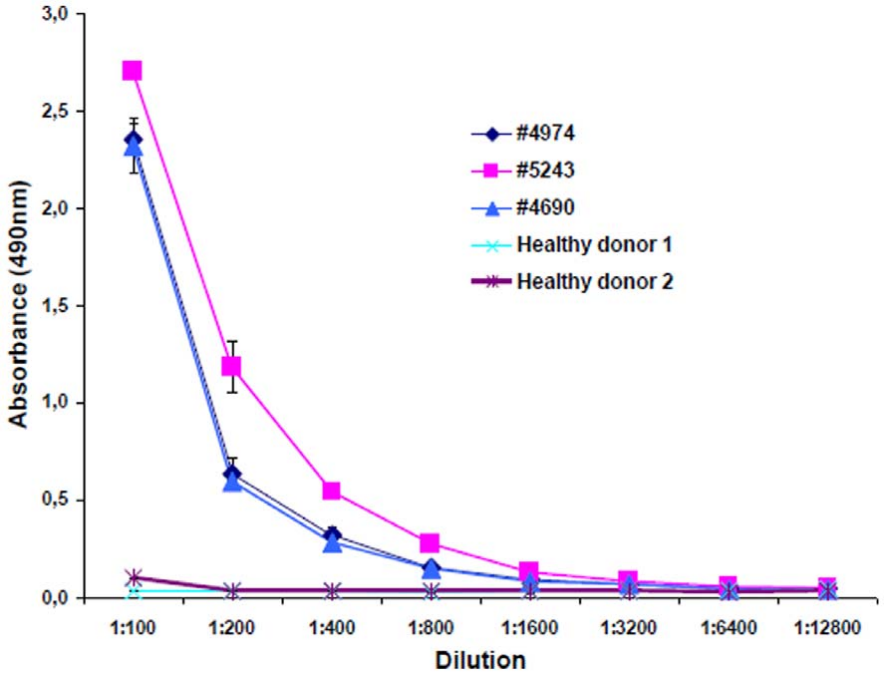
Titration of CD28 autoantibodies in the sera of patients and healthy donors measured by Elisa. Sera from three patients (#4974, #5243, #4690) and two healthy donors were titrated. The curves obtained from the healthy donors cannot be distinguished in this figure.

Antibody titres were high in all positive serum samples. Even at a serum dilution of 1∶12.800 antibodies were dectectable indicating their pathophysiological relevance (Figure 6).

### 9. Hepatitis Patients

CD28 abs were detected in 45 of 212 patients (21.2%). All patients received or had received interferons in the recent past because of hepatitis B or C virus infections. The occurrence of CD28 abs did neither correlate with therapy outcome (p = 0.4) nor with gender. The gender distribution of patients with CD28 abs (32 males, 13 females) did not differ from the total cohort of hepatitis patients.

### 10. CD28 and Interferon Therapy

CD28 abs occurred significantly more often in patients receiving interferons independent of the underlying disease (p<0.001, Chi square). In patients receiving interferons CD28 abs were detected in 19.9% of the cases, in patients who were not receiving interferons CD28 abs occurred only in 7.7% of the cases.

### 11. CD28 Autoantibodies have a Titer-dependent Effect on Jurkat T Cells

Jurkat T-cells are known to express the CD28 receptor and to be sensitive for stimulation with a mouse CD28 specific monoclonal antibody (clone 15E8). For this reason we used Jurkat cells for further experiments with our human CD28 serum autoantibodies.

**Figure 7:**
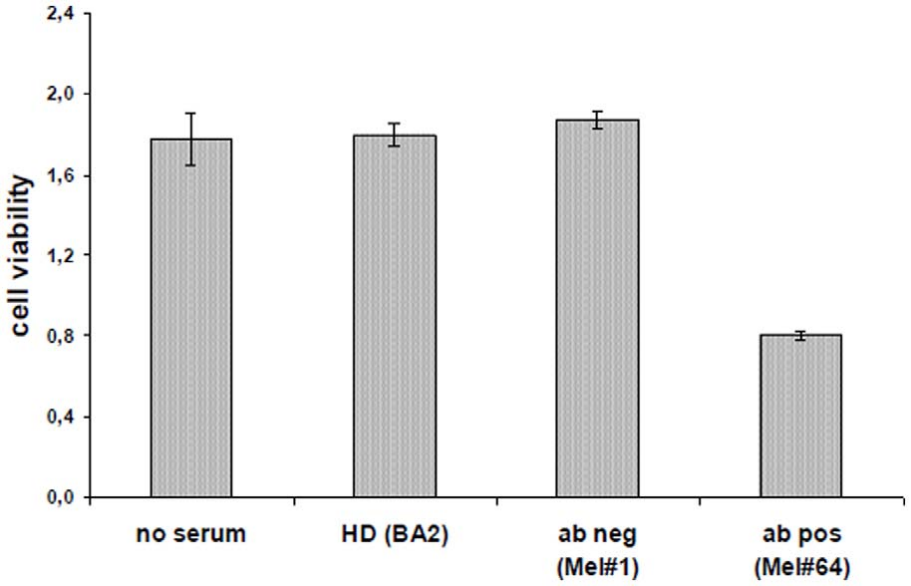
Human CD28 autoantibody containing sera have an effect on Jurkat T cell viability (EZ4U assay). Jurkat cells were incubated with sera derived from melanoma patients containing CD28 autoantibodies (Mel#64) or not (Mel#1) and from a healthy donor (HD). Jurkat cells without the addition of serum were used as control.

First, the effect of CD28 autoantibody containing sera on Jurkat cells was examinated. Cells were pre-incubated with serum, followed by stimulation with coated murine monoclonal anti-CD28 antibody (clone 15E8). In contrast to sera derived from healthy persons or from melanoma patients without CD28 autoantibodies, CD28 autoantibody containing sera showed an inhibitory effect on Jurkat cell stimulation (Fig. 7).

**Figure 8:**
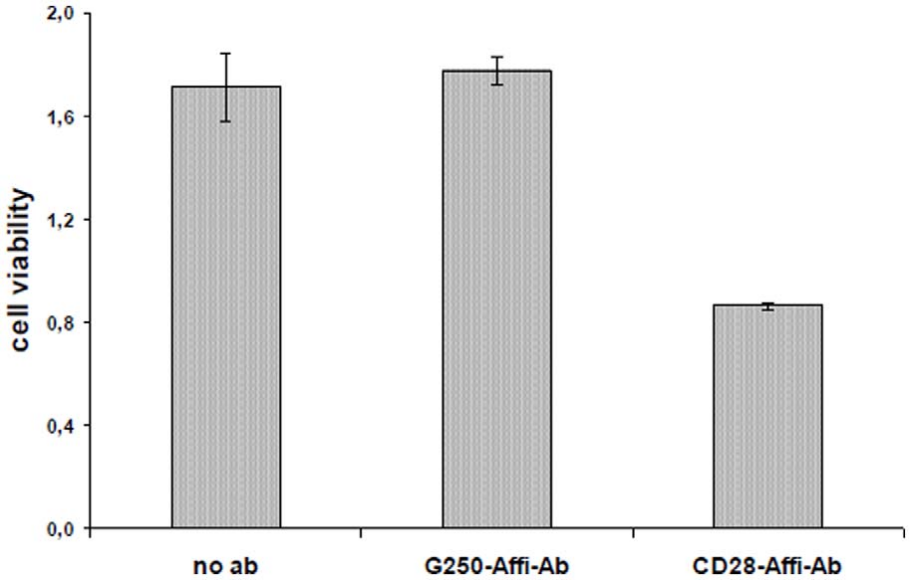
Purified human CD28 autoantibodies have an effect on Jurkat T cell viability (EZ4U assay). Jurkat cells were incubated with autoantibodies purified from patients serum by affinity chromatography. Purified CD28 autoantibodies show reduced cell viability while purified G250 autoantibodies have no effect on cell viability when compared with Jurkat cells not incubated with antibodies.

To verify that the inhibitory effect is derived from the CD28 autoantibody itself and not from other serum components, the CD28 abs were purified from human serum by CD28 affinity chromatography. We used purified human CD28 autoantibodies and compared their effect with a similar handled commercially produced chimeric mouse/human G250 antibody (mouse Fab_2_+ human Fc, kindly provided by Wilex Biotechnology GmbH, Munic, Germany)). We could prove an inhibitory effect on Jurkat T cell stimulation (Fig. 8).

**Figure 9:**
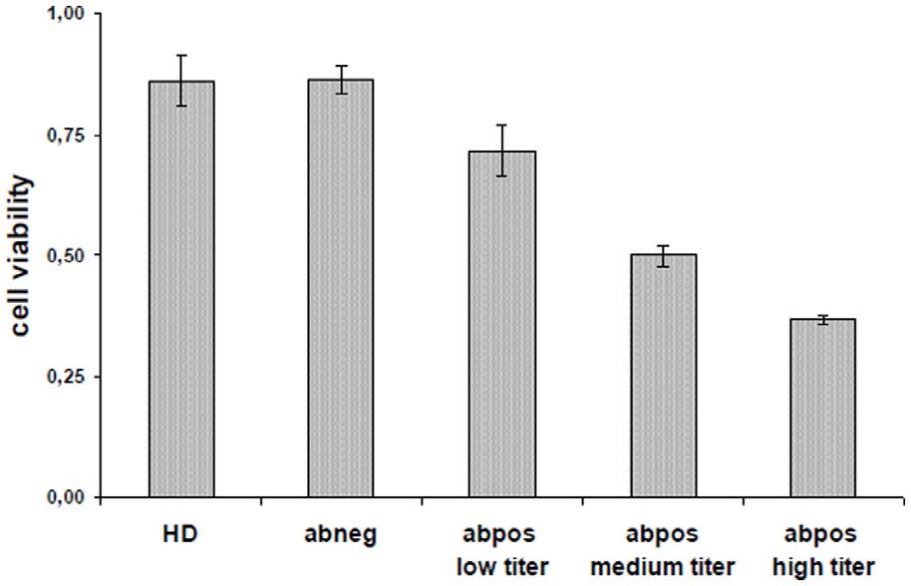
The inhibitory effect of human CD28 autoantibodoes is titer-dependent. Jurkat cells were incubated with sera derived from patient #64 that had been collected at different time points and that showed different titers of CD28 autoantibodies. Sera derived from a healthy donor (HD) or from a melanoma patients without CD28 autoantibodies were used as control. Cell viability was measured by EZ4U assay.

In additional experiments a clear relationship between the CD28 abs serum titer and the inhibitory effect on stimulated Jurkat cells was observed. This could be shown when serum of a patient (#64) was used who’s autoantibody titer had increased over time. A high CD28 abs titer resulted in a stronger inhibition than a low titer. Sera taken from healthy persons or sera from melanoma patients without CD28 autoantibodies did not have an inhibitory effect (Fig. 9).

**Figure 10:**
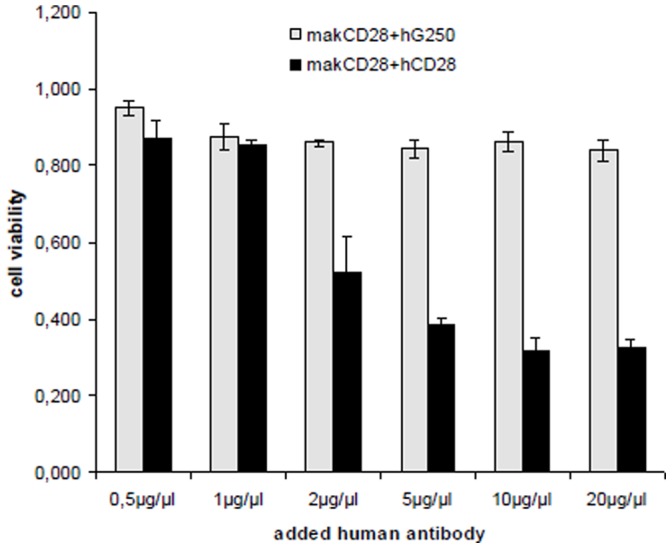
Competition assay using a mouse CD28 monoclonal antibody and purified human CD28 autoantibodies. Jurkat cells were incubated with mixtures of mouse monoclonal CD28 antibodies with increasing amounts of purified human autoantibodies (CD28 or G250). Competition was detected for human CD28 autoantibodies while human G250 autoantibodies had no effect. Cell viability was measured by EZ4U assay.

A competition assay using mouse monoclonal abs and purified human CD28 abs could further prove the direct inhibitory effect of human CD28 abs derived from melanoma patients on the CD28 receptor of Jurkat cells (Figure 10).

## Discussion

In the T-cell dependent immune response CD28 and CTLA-4 operate as opponents, CD28 as an accelerator and CTLA-4 as a brake-operator [3], [12].

During the last decade, the search for blocking or activating antibodies that address either CD28 or CTLA-4 had been a challenge for scientists worldwide.

In the past, Neuber *et al.* proved the existence of CD28 abs in patients with atopic diseases [4]. It was presumed that these antibodies stimulate T cells and may play an important role in chronic allergic inflammation as sera from patients with atopic dermatitis containing CD28 abs were able to stimulate T cell proliferation *in vitro* [4].

Basically, it is imaginable that inhibitory as well as stimulating CD 28 abs exist. In case of activation, CD28 abs binding could either lead to immune stimulation through activation of T-effector cells or immune suppression through stimulation of T regs. Inhibitory abs could lead to failed activation by sterical blocking of the interaction of the CD28 receptor with its physiologic ligands CD80/CD86. Activating effects can also be dose-dependent: Although monoclonal superagonistic CD28 antibodies “preferentially” activate Tregs, at higher doses, they also activate conventional T cells [13].

We found a significantly higher prevalence of CD28 abs in the group of melanoma patients and patients with viral hepatitis than in the other control groups investigated. This might be due to therapies with IFN as the prevalence of CD28 abs was significantly higher in patients receiving interferons (p<0.001) independent of the underlying disease. Hence, if IFN really abets the generation of CD28 abs, and the latter cause immune suppressive effects by stimulation of T regs or blocking of the CD28 receptor in its proper function, the continuation of IFN-therapies should be critically reconsidered in melanoma patients who develop CD28 abs. In fact, the risk of death due to melanoma was significantly increased in patients with CD28 abs. Hence, apart from its biological meaning anti-CD28 could become a valuable adjunct marker of progression.

Impaired IFN signalling has been observed in multiple sclerosis and chronic hepatitis C infection [14], [15]. Critchley-Thorne *et al.* identified defects in IFN signalling as a dominant mechanism of immune dysfunction in cancer patients [7], [8]. They demonstrated that a defect in type-I-IFN-signalling in T cells and B cells negatively impacts on the function of these cells. In the early phases of an immune response, IFNs act as a “third signal” required in addition to the first (antigen) and second (co-stimulation) signals for full activation and memory development rather than tolerance [7], [16]. Experiments of Critchley-Thorne and co-workers focused on a subgroup of melanoma patients in which low responses to type-I-IFN were observed. T-cells from low response patients exhibited functional abnormalities and lower survival following stimulation with anti-CD3 and anti-CD28 antibodies [8]. In such IFN low-response patients pre-existing or developing CD28 abs could be particularly harmful *in vivo* especially in patients with impaired IFN signalling. Therefore, the possibility must be considered that CD28 abs lead to a worse response to IFN treatment by hampering IFN signalling, although in our study occurrence of CD28 abs did not correlate with response to IFN therapy in melanoma or hepatitis patients.

Loss or inactivation of the CD28 receptor renders T lymphocytes unable to undergo clonal expansion and, in combination with increased expression of CD95, leads to an enhanced tendency to undergo apoptosis [17]. Urbaniak-Kujda *et al.* demonstrated that these conditions play a major role in the development of cutaneous T cell lymphoma [18]. In line with this hypothesis, Bouwhuis and co-workers found a reduced PFS in melanoma patients with polymorphisms of the CD28 gene [19]. These observations raise the question if CD28 serum abs in melanoma patients may have a similar effect by inhibiting or blocking the CD28 receptor in its proper function. Herein we collect preliminary evidence that CD28 abs of melanoma patients in fact have inhibitory effects on the CD28 receptor of Jurkat cells and lead to a titer-dependent reduced stimulation of Jukat cells *in vitro.* Further T-cell assays are warranted to investigate the role of CD28 serum abs on T-cells from melanoma patients. As this was a retrospective study we were not able to investigate T-cells from CD28 abs positive patients as the majority of the patients had died at the time of analysis.

If autoimmunity in melanoma patients is associated with prolonged survival or not has recently been discussed controversially.

Gogas and colleagues showed that autoimmunity - reflected by different serum autoantibodies such as antithyroid antibodies - in stage IIB, IIC or III melanoma patients under treatment with adjuvant high-dose interferon was associated with prolonged PFS and OS [20]. Stuckert *et al.* was able to present further evidence for this observation [21]. In our study neither the occurrence of anti-nuclear antibodies (ANA) nor thyreoglobulin antibodies (TG-abs) was associated with prolonged PFS or OS, nor did we found a correlation between occurrence of ANA or TG abs and the occurrence of CD28 abs in our melanoma patients. The latter indicates that these antibodies develop independently from each other.

Bouwhuis *et al.* investigated 220 melanoma patients in different adjuvant EORTC studies receiving IFN-alpha, who were antibody negative at baseline. Occurrence of autoantibodes during follow-up was higher in the patients treated with pegylated interferon (PEG-IFN) (18% in the observation arm, 52% in the PEG-IFN-arm) [19]. Autoantibody appearance was of prognostic importance using a model in which the so-called guarantee-time bias was disregarded. Guarantee-time bias means that patients with longer survival have a higher chance to develop auto-antibodies, whereas early relapses or early progression of disease is found more likely in antibody-negative patients because these patients experience relapse before auto-antibodies can develop. When guarantee-time bias was taken into account, a significant correlation was lost [19], [22]. The authors concluded that appearance of autoimmune antibodies is neither a prognostic nor a predictive factor for improved outcome in patients with melanoma treated with PEG-IFN. In further studies Bouwhuis *et al.* proved that autoimmune phenomena are more frequently observed in melanoma patients receiving immunotherapies, i.e. interleukin, interferon and anti-CTLA-4 treatment [23]. But considering the confounding factor guarantee-time bias, the association between an improved outcome in melanoma patients receiving IL-2 and autoimmunity was lost [23].

As another evidence that autoimmunity is not necessarily associated with prolonged survival in melanoma patients, we found no difference in progression-free or overall survival for melanoma patients with our without anti-retinal antibodies in a recent study in 2008, although a tendency for a shortened progress-free and overall survival for patients with anti-retinal antibodies could have been seen [24].

In conclusion it is likely that IFN therapy leads to the generation of CD28 abs. And it is not unlikely that this is linked to an increased death risk in melanoma patients. Hence CD28 abs could become a marker to determine a point in time when interferon therapy should better be stopped and intensive follow-up should be started to recognize a progression as soon as possible.

Further prospective studies are necessary to confirm our data and it is also highly interesting to analyze CD28 abs formation during the most recently invented immunotherapies with anti-CTLA.
